# Estimating restricted mean survival time and expected life-years lost in the presence of competing risks within flexible parametric survival models

**DOI:** 10.1186/s12874-021-01213-0

**Published:** 2021-03-11

**Authors:** Sarwar I. Mozumder, Mark J. Rutherford, Paul C. Lambert

**Affiliations:** 1 Biostatistics Research Group, Department of Health Sciences, University of Leicester, University Road, Leicester, LE1 7RH UK; 2 Department of Medical Epidemiology and Biostatistics, Karolinska Institutet, Stockholm, Sweden

**Keywords:** Competing risks, Restricted mean survival time, Restricted mean life time, Flexible parametric model, Life-years lost, Survival analysis

## Abstract

**Background:**

Royston-Parmar flexible parametric survival models (FPMs) can be fitted on either the cause-specific hazards or cumulative incidence scale in the presence of competing risks. An advantage of modelling within this framework for competing risks data is the ease at which alternative predictions to the (cause-specific or subdistribution) hazard ratio can be obtained. Restricted mean survival time (RMST), or restricted mean failure time (RMFT) on the mortality scale, is one such measure. This has an attractive interpretation, especially when the proportionality assumption is violated. Compared to similar measures, fewer assumptions are required and it does not require extrapolation. Furthermore, one can easily obtain the expected number of life-years lost, or gained, due to a particular cause of death, which is a further useful prognostic measure as introduced by Andersen.

**Methods:**

In the presence of competing risks, prediction of RMFT and the expected life-years lost due to a cause of death are presented using Royston-Parmar FPMs. These can be predicted for a specific covariate pattern to facilitate interpretation in observational studies at the individual level, or at the population-level using standardisation to obtain marginal measures. Predictions are illustrated using English colorectal data and are obtained using the Stata post-estimation command, standsurv.

**Results:**

Reporting such measures facilitate interpretation of a competing risks analysis, particularly when the proportional hazards assumption is not appropriate. Standardisation provides a useful way to obtain marginal estimates to make absolute comparisons between two covariate groups. Predictions can be made at various time-points and presented visually for each cause of death to better understand the overall impact of different covariate groups.

**Conclusions:**

We describe estimation of RMFT, and expected life-years lost partitioned by each competing cause of death after fitting a single FPM on either the log-cumulative subdistribution, or cause-specific hazards scale. These can be used to facilitate interpretation of a competing risks analysis when the proportionality assumption is in doubt.

## Background

In observational studies of time-to-event data, researchers are often interested in decomposing the overall probability of death into component parts due to the event of interest, and competing, but mutually exclusive outcome events. For example, in cancer studies, it is of interest to partition the overall probability of death into the probability of death due to cancer and the probability of death due to other causes. These are referred to as cause-specific cumulative incidence functions (CIFs) and are often chosen as the primary estimand of interest. The cause-specific CIF gives the probability of dying from the cause of interest at a particular time whilst also being at risk of dying from other causes of death [1, 2]. In order to arrive at these quantities and to circumvent bias, methods that appropriately account for the competing nature of the events must be applied. The restricted mean failure time (RMFT) has been proposed as an alternative summary measure that is based on the area under the all-cause probability of death up to a specific time-point[3]. In an analogous way to the decomposition into cause-specific CIFs, the RMFT can be further partitioned to give the expected number of life years lost due to a specific cause before a given time-point. In this paper, we describe how the aforementioned measures can be obtained using a flexible parametric model (FPM) as the estimation approach by modelling covariate effects either using (1) the direct relationship with the cause-specific CIF on the subdistribution hazards (SDHs) scale, or (2) modelling all cause-specific hazard functions (CSHs) to obtain each cause-specific CIF [4–7]. Choosing FPMs as the estimation method allows us to estimate effects conditional on covariates, and effects averaged over specific covariate distributions.

Forming contrasts to compare exposure groups is often a further key focus in many large population-based studies. A common approach would be to report either cause-specific hazard ratios (HRs), which measures the effect of an exposure group on the rate of dying from a cause of interest, or sub-distribution hazard ratios (SHRs), which measures the effect of an exposure group on the risk of dying from a cause of interest, whilst assuming that the cause-specific HR or SHR was constant over time. However, it is well known, for instance, that the HR for tumor size in cancer studies will vary over time since diagnosis, with stronger relative effects shortly after diagnosis [8–10]. When non-proportional hazards are present i.e. when the HR is expected to change over time, it has been argued that the HR as the target estimand is not appropriate and there are further issues in making causal inferences using HR measures due to its non-collapsibility as a relative risk measure [11]. As an alternative to the HR, estimation of the difference in restricted mean survival time (RMST), also known as the restricted mean lifetime (RMLT), as the primary estimand has been proposed [12–19]. This, in contrast to the HR, is known as a collapsible measure [11, 20]. Furthermore, this single summary measure can still be presented when relaxing the assumption of proportional hazards within the model-building process. These can either be presented as conditional differences, which is the average covariate effect on the individual, or marginal differences, which is the average covariate effect on the population [21].

In the presence of competing risks, Andersen [3] introduces the analogue to the RMST measure for the CIF which gives the (total) number of years lost before a pre-specified time, i.e. RMFT, and demonstrates how this can be partitioned to give the expected number of life-years lost due to each cause of death [22]. In his approach, he estimates RMFT and expected number of life-years lost using regression models with pseudo-observations [3, 23]. These models only allow prediction for specific quantities of interest and only at single time-points. Therefore separate models must be fitted to estimate, for example, either the cause-specific CIF or RMFT, when it may be of interest to obtain both and at various time-points. For instance, to allow comparability and to obtain the entire picture of the impact of different groups on outcome, it has been suggested that differences in RMST, RMFT and therefore, expected number of life-years lost, should be reported alongside their respective survival, or cumulative incidence functions [24]. Alternatively, the Royston-Parmar FPM approach for estimating RMST, which is extended for competing risks to estimate partitioned RMFT, as introduced in this paper, can be used [25]. In contrast to more popular approaches, such as the Cox model, a parametric estimate of the baseline hazard function is obtained as part of the full likelihood function. This is estimated using restricted cubic splines (RCS), allowing easy prediction of absolute comparisons between key quantities of interest. What’s more, standard errors for predictions can be estimated via the delta method, which offers computational advantages in larger data compared to approaches for non-parametric and semi-parametric methods which use bootstrapping, or jack-knife resampling methods [26]. Further advantages include the easy inclusion of time-dependent effects using interactions with RCS for relaxing the proportional hazards assumption. Estimating both the baseline effects, and time-dependent effects to model departures from the baseline using splines allows a unified approach for estimating all required parameters in order to obtain predictions of all quantities of interest. Therefore, we introduce in this paper how RMFT as the chosen estimand can be estimated using FPMs in the presence of competing risks on either the CSHs or cumulative incidence scale as the estimator [5, 7]. This extends on previous work by Royston and Parmar where estimation in the presence of competing risks is not considered [16]. This approach allows the researcher to obtain differences in effect between exposure groups either conditional on a set of covariates, or averaged over a covariate distribution, also known as marginal estimates. Furthermore, both marginal and conditional estimates can be obtained from the same model where the prediction of marginal estimates using standardisation is proposed [27, 28]. We, therefore, further demonstrate how difference in marginal estimates of RMFT as the chosen estimand for the comparison between covariate groups can be obtained within FPMs for competing risks.

We begin with a brief review of competing risks in the Overview of competing risks section and highlight particular interest in the cause-specific CIF. This is followed by an introduction of RMFT as the chosen estimand in Overview of restricted mean survival time for competing risks section along with other useful measure such as expected life-years lost. The Flexible parametric survival models section details FPM approaches for estimation in the presence of competing risks. In the Estimation section, we show how absolute differences between RMFT and expected number of life-years lost are calculated to assess the impact of a covariate. We further demonstrate how these models can be used for easily obtaining marginal estimates and associated contrasts using standardisation in the Standardisation for marginal differences section. For illustration of these various measures, English colorectal cancer data obtained from National Cancer Registration and Analysis Service (NCRAS) is analysed in the Results: colorectal cancer survival in England section where comparisons between the most and least deprived colorectal cancer patients are made, accompanied by Stata code for estimation in Appendix: Stata code for obtaining predictions. Finally, the paper is concluded with a discussion on the use and estimation of RMST in the presence of competing risks within FPMs. Although we specifically consider application to cancer studies, where the event of interest is death from cancer, the methods are generalizable to other time-to-event data and therapeutic areas.

## Methods

### Overview of competing risks

In the presence of competing risks, an individual is at risk of failing from more than one event where the occurrence of one event means that others cannot occur. In the context of a cancer survival study, this is when a patient can die from a multitude of other causes as well as the cancer itself. However, if the patient dies from one of these other causes, it means that the time at which the patient would have died from cancer is never observed. One of the key quantities, and often the chosen estimand of interest within this framework, is the cause-specific CIF [1].

#### Cause-specific CIF

Let *T* be a non-negative random variable for the time to death from any cause. Furthermore, let *D* denote the cause of death in the presence of *k*=1,…,*K* competing risks, where *D*=1,…,*K*. It follows that the estimand, cause-specific CIF, *F*_*k*_(*t*), is defined as,

1 $$ F_{k}(t) = P(T < t, D = k)  $$

This is interpreted as the probability of dying from cause *k* by time *t* whilst also being at risk of dying from other competing causes of death. Note here that the cause-specific CIF is an improper distribution function since the integral of *F*_*k*_(*t*) at infinity is always less than 1 [3].

The target estimand, the cause-specific CIF, can be calculated using either all *k* CSH functions, or by utilising the one-to-one relationship between the cause-specific SDH function. These are briefly introduced below.

#### Cause-specific hazards

The CSHs, $h^{cs}_{k}(t)$, give the instantaneous mortality rate from a particular cause *k* given that the patient is still alive at time *t* in the presence of all the other causes of death such that,

2 $$  h^{cs}_{k}(t) = {\lim}_{\Delta t\to 0 }\frac{P[t \leq T < t + \Delta t, D = k | T \geq t)}{\Delta t}  $$

It follows that the target estimand, the cause-specific CIF, can be calculated as a function of all *k* CSH functions,

3 $$ F_{k}(t) = \int^{t}_{0} S(u)h^{cs}_{k}(u) \text{d}u  $$

where $S(t) = \exp {\left (-\sum _{k=1}^{K} \int _{0}^{t} h_{k}^{cs}(u)\text {d}u\right)}$ is the all-cause survival function.

#### Subdistribution hazards

Alternatively, Gray [29] introduces the SDH function for cause $k, h_{k}^{sd}(t)$, which offers a direct one-to-one relationship with the cause-specific CIF estimand. This has the following mathematical formulation,

4 $$ \begin{aligned} h_{k}^{sd}(t) = {\lim}_{\Delta t\to 0 }\frac{P[t \leq T < t + \Delta t, D = k | T \geq t \cup (T < t \cap D \neq k)}{\Delta t} \end{aligned}  $$

which is interpreted as the instantaneous “sub”-rate of failure at time *t* from cause *k* amongst those who are still alive, or have died from any of the other *K*−1 competing causes excluding cause *k* [30].

This is not defined as a typical epidemiological rate since the risk-set includes those that are either still alive *or* have died from a competing cause of death. However, if individuals do not experience the competing event, then the SDH rate and the CSH rate are both equivalent [31]. It should be noted that, due to the nature of the risk-set in the definition of a SDH, it is very difficult to interpret [30, 32, 33].

The cause-specific CIF estimand can be directly obtained from the SDH for cause *k* using the standard survival transformation of the cumulative SDH function for cause $k, H^{sd}_{k}(t)$, such that,

5 $$ F_{k}(t) = 1 - \exp{\left[-H^{sd}_{k}(t)\right]}  $$

This shows that a one-to-one correspondence is maintained between the SDH function for a specific cause of death and the cause-specific CIF.

The choice of which scale to model on depends entirely on the research question to be answered which would relate to other quantities specific to the modelling approach that may be of interest. For instance, if primary interest is in aetiological outcome, then the estimand of interest would be on the CSH rates. For interest in prognostic outcome, one may wish to quantify effects on the risk of dying from a specific cause of death. In this case, the estimand of interest would be the cause-specific CIF, which can be obtained as function of all CSHs, or through the SDH for cause *k*. Further discussion on this topic is provided elsewhere [4, 34].

### Overview of restricted mean survival time for competing risks

The RMST measure quantifies the average survival, or time lived, of a patient from time 0 up to a pre-defined time-point, *t*^∗^. In the absence of competing risks, the RMST before *t*=*t*^∗^,*μ*(*t*^∗^), of a random variable *T* is equal to the expectation of min(*T*,*t*^∗^). RMST, in the absence of covariates, can be expressed as the estimand,

6 $$ \mu(t^{*}) = E(\min(T, t^{*})) = \int_{0}^{t^{*}} S(u) \text{d}u  $$

where *S*(*t*) is the all-cause survival function. If time is measured in years, this is the average life-years lived before time *t*^∗^. The choice of *t*^∗^ should be pre-determined and clinically motivated, and will vary by, for example, cancer types [15, 16]. This is also often chosen at maximum follow-up time [13, 35].

In addition to this, Andersen [3] proposes calculation of the expected number of years lost before time *t*^∗^ such that the estimand can be defined as,

7 $$ L(0, t^{*}) = t^{*} - E(\min(T, t^{*})) = t^{*} - \int_{0}^{t^{*}} S(u) \text{d}u  $$

#### Expected loss in life due to a cause of death

In the presence of competing risks, Andersen [3] shows that the (total) number of years lost, *L*(0,*t*^∗^), can be decomposed into the number of years lost due to each cause *k* [22]. It follows that since,

8 $$ S(t) = 1 - \sum_{k=1}^{K} F_{k}(t)  $$

then the RMST in Eq. 6 can be expressed as a function of each cause-specific CIF through the following integral,

9 $$ \begin{aligned} \mu(t^{*}) = E(\min(T, t^{*})) &= \int_{0}^{t^{*}} S(u) \text{d}u = \int_{0}^{t^{*}} 1 - \sum_{k=1}^{K} F_{k}(u) \text{d}u \\ &= t^{*} - \int_{0}^{t^{*}} \sum_{k=1}^{K} F_{k}(u) \text{d}u \end{aligned}  $$

Equation 7 can also be written as a sum of the integral of each cause-specific CIF such that,

10 $$ L(0, t^{*}) = t^{*} - \int_{0}^{t^{*}} S(u) \text{d}u = \sum_{k=1}^{K} \int_{0}^{t^{*}} F_{k}(u) \text{d}u  $$

which may also be referred to as restricted mean failure time (RMFT). It follows that RMFT can be partitioned where we have the estimand,

11 $$ L_{k}(0, t^{*}) = \int_{0}^{t^{*}} F_{k}(u) \text{d}u  $$

which gives the expected number of years lost due to cause *k* before time *t*^∗^.

### Flexible parametric survival models

For competing risks data, many adopt the cause-specific Cox proportional hazards model, or the Fine & Gray approach as the chosen estimator for the estimands introduced in the Overview of competing risks and Overview of restricted mean survival time for competing risks sections. Here, we propose the use of FPMs as the chosen estimator in order to obtain the estimand of interest. FPMs are increasing in popularity since the baseline SDH or CSH function is estimated as part of a fully specified likelihood function and allows the estimation of various estimands from a single model [5, 7]. These models were introduced for standard survival data (in the absence of competing risks) on various scales by Royston and Parmar [9] using a general link function, *g*(·), to better capture and represent the behaviour of real world data. To increase flexibility and more accurately capture complex shapes of the cumulative hazard function, Royston and Parmar [9] proposed the use of RCS (see Appendix A). Under the assumption of proportional hazards, Rutherford et. al [36] showed in simulations that FPMs more accurately capture complex shapes of hazard functions. They further illustrated that unbiased estimates of the HRs were obtained. Given a vector of *M* knots, **m**, and a vector of *M*−1 parameters, *γ*, with a RCS function, *s*(ln(*t*);*γ*,**m**) we have that,

12 $$ \eta = g(G_{k}(t \mid \mathbf{x}_{k})) = s_{k}(\ln(t); \pmb{\gamma}_{k}, \mathbf{m}_{k}) + \mathbf{x}_{k}\pmb{\beta}_{k}^{T}  $$

where, **β**, is a vector of co-efficient parameters and, **x**, is a vector of covariates.

Equation 12 can also be easily extended for time-dependent effects to model non-proportionality by fitting interactions between the associated covariates and the spline functions. Using this interaction, a new set of knots, **m**_*e*_, are introduced, which represent the *e*^*th*^ time-dependent effect with associated parameters *α*_*e*_. If there are *e*=1,⋯,*E* time-dependent effects, Eq. 12 can be extended such that,

13 $$ {}\begin{aligned} \eta &= g(G_{k}(t \mid \mathbf{x})) = s(\ln(t); \pmb{\gamma}, \mathbf{m}_{0}) + \mathbf{x}\pmb{\beta}^{T} \\&\quad+ \sum_{l=1}^{E}s(\ln(t); \pmb{\alpha}_{l}, \mathbf{m}_{l})x_{l} \end{aligned}  $$

Non-proportional hazards are a common occurrence in studies with long follow-up time, or, in the context of cancer studies, when the effect of covariates (e.g tumor size, or treatment) on cancer-related mortality varies over time [8–10, 19]. FPMs, extended for time-dependent effects as in the Equation above, have also been shown to accurately capture complex shapes of the hazard function with time-dependent effects i.e. where there is non-proportionality in the hazards [37]. This result is consistent with what was shown by Rutherford et. al. for FPMs without time-dependent effects i.e. proportional hazards, as mentioned above [36]. Further technical details on FPMs for standard survival data in the absence of competing risks can be found elsewhere [9, 25, 38].

The models described in Eqs. 12 and 13 can be fitted on either CSHs scale [7], where *G*_*k*_(*t*∣**x**)=*S*_*k*_(*t*∣**x**), or cumulative incidence scale [5, 6], where *G*_*k*_(*t*∣**x**)=1−*F*_*k*_(*t*∣**x**), based on different link functions, *g*(·). The relationship of these with the cause-specific CIF are defined in the Cause-specific hazards and Subdistribution hazards sections. Therefore, it follows that, using a complementary log-log link function, the corresponding log-cumulative CSHs FPM (otherwise referred to as a cause-specific FPM), is,

14 $$ {}\begin{aligned} \eta_{k}^{cs} &= \log \left[-\log \left[S_{k}\left(t \mid \mathbf{x}_{k}\right)\right]\right]=\log \left[H_{k}^{cs}\left(t \mid \mathbf{x}\right)\right]\\ &\quad= s_{k}(\ln(t); \pmb{\gamma}_{k}, \mathbf{m}_{k}) + \mathbf{x}_{k}\pmb{\beta}_{k}^{T} \end{aligned}  $$

and can be fitted in a similar way to the standard FPM. Alternatively, models for all *k* causes can be fitted simultaneously by restructuring the data as described by Hinchliffe et. al. [7].

The log-cumulative SDHs FPM for cause *k* (also known as the flexible parametric cumulative incidence model, or FPCIM), on the other hand is defined as,

15 $$ {}\begin{aligned} \eta_{k}^{sd} &= \log \left[-\log \left[1-F_{k}\left(t \mid \mathbf{x}_{k}\right)\right]\right]=\log \left[H_{k}^{sd}\left(t \mid \mathbf{x}\right)\right] \\&\quad= s_{k}(\ln(t); \pmb{\gamma}_{k}, \mathbf{m}_{k}) + \mathbf{x}_{k}\pmb{\beta}_{k}^{T} \end{aligned}  $$

and can be fitted using the approach outlined using either the full likelihood function as described by Mozumder et. al. [5] or by using time-dependent censoring weights, similar to the Fine-Gray model, as detailed by Lambert et. al. [6]. As previously mentioned, alternative link functions are also available for models on either scale. See for example, Lambert et. al. [6].

### Estimation

#### Cause-specific cumulative incidence function

If modelling on the cumulative incidence scale using SDHs, after fitting the FPCIM in Eq. 15, the cause-specific CIF is obtained by the following,

16 $$ \widehat{F}_{k}\left(t \mid \mathbf{x}\right)=1-\exp \left(-\exp \left(\hat{\eta_{k}^{sd}}\left(t \mid \mathbf{x}\right)\right)\right)  $$

Alternatively, when modelling on the CSHs scale, after fitting the cause-specific FPM in Eq. 14, and as shown in Eq. 3, the integral below must be evaluated in order to obtain the cause-specific CIF,

17 $$ \widehat{F}_{k}\left(t \mid \mathbf{x}\right)=\int_{0}^{t} \widehat{S}\left(u \mid \mathbf{x}\right)\widehat{h}^{cs}_{k}\left(u \mid \mathbf{x}\right) du  $$

where the predicted CSH function is,

18 $$ \widehat{h}_{k}^{cs}\left(t \mid \mathbf{x}\right)= \frac{d s\left(\log (t) \mid \gamma, \mathbf{m}_{0}\right)}{d t} \exp \left(\eta_{k}^{cs}(t)\right)  $$

and the predicted all-cause survival function is,

19 $$ \widehat{S}\left(u \mid \mathbf{x}\right) = \prod_{k=1}^{K} \exp \left(-\int_{0}^{t} \widehat{h}_{k}^{cs}(u \mid \mathbf{x}) \mathrm{d} u\right)  $$

However, as the above integral is not of closed form, numerical approximation techniques must be used. Here, the Gauss-Legendre quadrature approximation method is used [39]. Details of this method is provided in Appendix B. Therefore, after fitting the cause-specific FPM for each *k* causes, the predicted cause-specific CIF at *t*_1_,⋯,*t* different time-points over an interval [0,*t*] is approximated by applying Gaussian quadrature rules with *W*(*u*)=1 such that,

20 $$ \begin{aligned} \widehat{F}_{k}\left(t \mid \mathbf{x} \right)=\int_{0}^{t} f_{k}^{*}(u) \mathrm{d}u \approx \frac{t-0}{2} \sum_{i=1}^{m} w_{i}^{\prime} f_{k}^{*}\left(\frac{t-0}{2} u_{i}^{\prime}+\frac{t+0}{2}\mid \mathbf{x} \right) \end{aligned}  $$

where, $\widehat {f}_{k}^{*}(t)$, is the “sub”-density function such that,

21 $$ \widehat{f}_{k}^{*}\left(t \mid \mathbf{x}\right)=\widehat{S}\left(t \mid \mathbf{x}\right)\widehat{h}^{cs}_{k}\left(t \mid \mathbf{x}\right)  $$

#### Restricted mean failure time and expected number of life-years lost due to each cause of death

If RMFT is the chosen target estimand of interest, this can be predicted as the integral under the all-cause CIF such that,

22 $$ \widehat{L}\left(0, t^{*}\right) = \int_{0}^{t^{*}} \sum_{j=1}^{K} \widehat{F}_{j}(u) \mathrm{d} u=\sum_{j=1}^{K} \int_{0}^{t^{*}} \widehat{F}_{j}(u) \mathrm{d} u  $$

where the predicted expected number of life-years lost before time *t*^∗^ due to each cause *k* is,

23 $$ \widehat{L}_{k}\left(0, t^{*} \mid \mathbf{x} \right)=\int_{0}^{t^{*}} \widehat{F}_{k}(u \mid \mathbf{x}) \mathrm{d}u  $$

Again, as above in Eq. 17, as the integral is of closed-form, we use the Gauss-Legendre quadrature approximation technique to numerically evaluate,

24 $$ {}\int_{0}^{t^{*}} \widehat{F}_{k}(u) \mathrm{d}u \approx \frac{t^{*}-0}{2} \sum_{i=1}^{m} w_{i}^{\prime} \widehat{F}_{k}\left(\frac{t^{*}-0}{2} u_{i}^{\prime}+\frac{t^{*}+0}{2} \mid \mathbf{x} \right)  $$

It follows that the RMST can also be obtained by,

25 $$ \widehat{\mu}\left(t^{*} \mid \mathbf{x} \right) = t^{*} - \sum_{j=1}^{K} \widehat{L}_{j}\left(0, t^{*}\right)  $$

#### Conditional differences

In population-based studies, i.e. non-randomised studies, it may be of interest to make absolute or relative comparisons between different covariate groups. As an alternative summary measure, or estimand, to the HR, we can calculate the difference in RMST between two covariate groups, or the difference in expected loss in life due to different causes [19]. Let *X* be a binary covariate that denote the group of interest and *Z* be the set of measured covariates with a specific covariate pattern **z**_*j*_. To estimate the average number of life years gained in group *X*=0 compared to group *X*=1, we have that,

26 $$ \hat{\mu}(t^{*} \mid X = 1, Z = \mathbf{z}_{j}) - \hat{\mu}(t^{*} \mid X = 0, Z = \mathbf{z}_{j})  $$

Alternatively, we can also estimate the expected reduction in the loss (or gain) in life due to cause *k* by,

27 $$ \hat{L}_{k}(0, t^{*} \mid X = 1, Z = \mathbf{z}_{j}) - \hat{L}_{k}(0, t^{*} \mid X = 0, Z = \mathbf{z}_{j})  $$

Partitioning in this way is particularly useful if covariates act differently on different causes of death. For example, those from a particular covariate group may lose (or gain) some life-years due to a specific cause of death in comparison to another covariate group.

Absolute measures of gains or losses in years of life are presented above as potential estimates of interest. To obtain relative measures, the ratio between the RMST estimates, or expected loss in life due to cause *k* for the two covariate groups are calculated. Extension can also be made for comparisons on a unit increase in a continuous covariate *Z*, and for time-dependent effects.

#### Standardisation for marginal differences

Regression standardisation is part of the estimator that can be used to obtain marginal predictions for different covariate groups at each observation given a set of measured confounders [27, 28]. Here, we apply standardisation to RMST and cause-specific CIFs estimates obtained from a flexible parametric competing risks survival model. In this case, it is of interest to compare the average life-years lived before time *t*^∗^ between two different groups [17, 18]. This is done by obtaining marginal estimates which are calculated as an average over every individual in the observed dataset. This enables comparisons that solely focus on the differences between the two groups of interest by forcing the same covariate distribution over multiple confounders. If all exposures and confounders are measured at baseline, this is essentially equivalent to the G-formula [40]. For example, to compare males and females, estimates must be standardised by age in order to force the same age distribution for both males and females. Extension can be made for multiple covariates and other potential confounders. This is calculated using an average of RMST estimates for each patient to summarise the risk for a certain covariate group. For instance, let *X* be an indicator variable that denotes the group of interest and *Z* be the set of measured covariates. Then the predicted RMST estimate for the *i*^*th*^ individual, where *i*=1,…,*N*, is,

28 $$ \widehat{\mu}_{i} = t^{*} - \int_{0}^{t^{*}} \sum_{k=1}^{K} \left[ \widehat{F}_{k}(u \mid X = x, Z = z_{i}) \right] \text{d}u  $$

where *X* is fixed to a specific value, *x*, and *Z* is the observed covariate pattern, *z*_*i*_, for the *i*^*th*^ individual. We can then average over the marginal distribution of *Z* for all the predicted restricted mean life estimates obtained for each individual *i* such that,

29 $$ E(\widehat{\mu}^{stand} \mid X = x, Z) = \frac{1}{N} \sum_{i=1}^{N} \widehat{\mu}_{i}  $$

This allows us to calculate marginal differences between covariate groups. For example, between group *X*=0 and group *X*=1, the marginal difference in RMST is,

30 $$ E(\widehat{\mu}^{stand} \mid X = 1, Z) - E(\widehat{\mu}^{stand} \mid X = 0, Z)  $$

In recent literature, some have advocated the use of RMST as a causal measure [41, 42]. For a causal interpretation, the consideration of additional assumptions are required and by adjusting for all appropriate confounders, these measures can be extended and interpreted as causal effects and thus, used as an estimand [21]. This is because, as shown above, they provide marginal comparisons averaged over the same covariate distribution by using standardisation. Standardisation, otherwise referred to as G-computation, has also been highlighted by Gran et al. [43] as an approach for obtaining useful summary causal-effect measures in more complicated multi-state models. However, this is beyond the scope of the paper and estimation of causal effects are not explicitly discussed here. Note also that we only consider time-fixed confounders and that there are additional complexities when considering time-dependent risk-groups [44].

## Results: colorectal cancer survival in England

### Data

Data was obtained from the National Cancer Registration and Analysis Service (NCRAS) to illustrate the estimation of various measures introduced in the Overview of restricted mean survival time for competing risks section. The data consist of English colorectal (ICD10: C18, C19 and C20) male and female cancer patients aged between 45 and 90 years old. Patients are diagnosed on or after 1998 are included with follow-up restricted to either 10 years or censored at 31 Dec 2013, whichever comes first. Analysis is further restricted to patients from the most or least deprived groups as defined by the upper and lower quintiles of the English index of multiple deprivation 2010 (IMD 2010). These groups are selected to simplify analysis and to make for easy illustration of presenting different metrics to allow comparisons between the two groups. The final data consisted a total of 159,022 individuals of which 48,845 die from cancer, 7,987 from cardiovascular disease (CVD) and 32,133 from other causes. In Appendix C, summary statistics on the age distribution, and number of patients in each deprivation and sex groups are provided.

### Model

For demonstration purposes, predictions are obtained after fitting an FPCIM simultaneously for all *k* causes of death and standard errors for confidence intervals (CIs) are obtained using the delta method. However, predictions are also available after fitting cause-specific FPMs. This paper focusses on the various estimands we can obtain from such models, namely, the RMST measure and expected life-years lost.

Models are fitted simultaneously for all *k* causes of death using the approach of Lambert et al. [6] and Geskus [45]. This fits the model after restructuring the data and applying time-dependent weights that are obtained parametrically to the censoring distribution of the competing causes of death. Alternatively, using the approach described by Jeong and Fine [46], models can be fitted on individual-level data using the full likelihood function [47]. Models for each of the causes of death include sex, IMD 2010 deprivation group (upper and lower quintile only) and a non-linear effect of continuous age using RCS with 3 DF centred at 45 years old at diagnosis. Time-dependent effects to relax the proportionality assumptions are included for sex, non-linear age and deprivation group with 2 DF and 3 DF are used for the baseline RCS function. In order to evaluate whether assuming non-proportional (subdistribution) hazards was more sensible, and is more consistent with the data, a likelihood ratio test was performed. This compared the FPCIM with time-dependent effects to relax the proportionality assumption to the one without that assumed proportional SDHs. The likelihood ratio test statistic was 752.94 and the associated p-value was less than 0.0001. This shows that relaxing the proportionality assumption leads to a statistically significant improvement in model fit. Note that this is an illustrative model and we therefore omit formal evalutation of the model performance. When evaluating the model in practice, we recommend conducting a sensitivity analysis, particularly in the selection of the number of knots. This can be done by comparing the Akaike information criterion and the Bayesian information criterion as an informal guide to selecting the appropriate number of knots and covariates [6].

### Analysis of data with conditional estimates

#### Cause-specific cumulative incidence functions

**Fig. 1:**
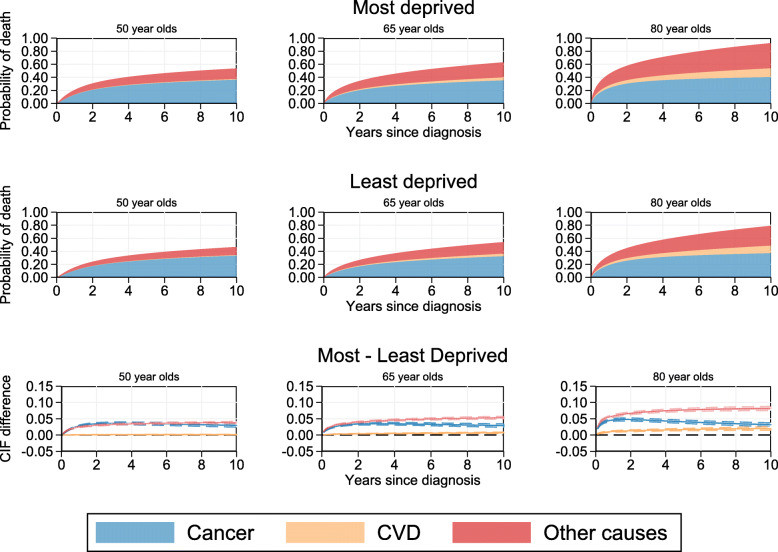
Stacked cause-specific CIFs by deprivation group and CIF differences for male patients at specific ages

Cause-specific CIFs are presented in Fig. 1 for male colorectal cancer patients. The probability of dying from cancer at 10 years from diagnosis for the most deprived male patients is approximately 36.5% (95% CI: 35.5%, 37.5%) for those aged 50 years old at diagnosis. This slightly increases to approximately 40.5% (95% CI: 39.8%, 41.1%) for those aged 80 years old at diagnosis. However, the largest change is in the probability of dying from other causes and CVD which have an increasing contribution to the probability of dying from any cause for older male patients from the most (and least) deprived groups. For instance, the probability of dying from any cause by 10 years from diagnosis for the most deprived 50 year old male patients at diagnosis is 53.6% of which 17.1% is due to other causes and CVD. In contrast, the all-cause probability of death for the most deprived male patients aged 80 years old diagnosis is much higher at 92.5%. However, although the probability of dying due to cancer has only increased from 36.5% to 42.5%, much of the overall probability of dying is due to other causes (38.4%) and CVD (13.6%).

Absolute CIF differences between the most and least deprived male patients aged 50, 65 and 80 years old at diagnosis are presented on the third row of Fig. 1. This shows that, for 50 year olds, the difference between CIFs for the most and least deprived groups are similar for deaths due to cancer and other causes. There is very little difference between the two deprivation groups for deaths due to CVD, however, this is due to a generally very low probability of death due to CVD. On the other hand, for older male patients, the difference in the probability of dying from other causes and CVD between the most and least deprived is larger and increases over time. This leads to a greater disparity in the probability of dying from other causes and CVD between the most and least deprived patients compared to the difference in the probability of dying due to cancer. Furthermore, after approximately 1 year from diagnosis for 65 year olds, and 2 years for 80 year olds, the difference in the probability of dying due to cancer for the most deprived compared to the least deprived patients reduces. This change in difference between the most and least deprived is greatest for the 80 year old male patients with cancer-specific CIF difference reducing from approximately 4.6% (95% CI: 4.2%, 5.0%) at 1 year from diagnosis to 3.2% (95% CI: 2.6%, 3.7%) by 10 years from diagnosis.

#### Restricted mean failure time and expected number of life-years lost due to a particular cause of death

**Fig. 2:**
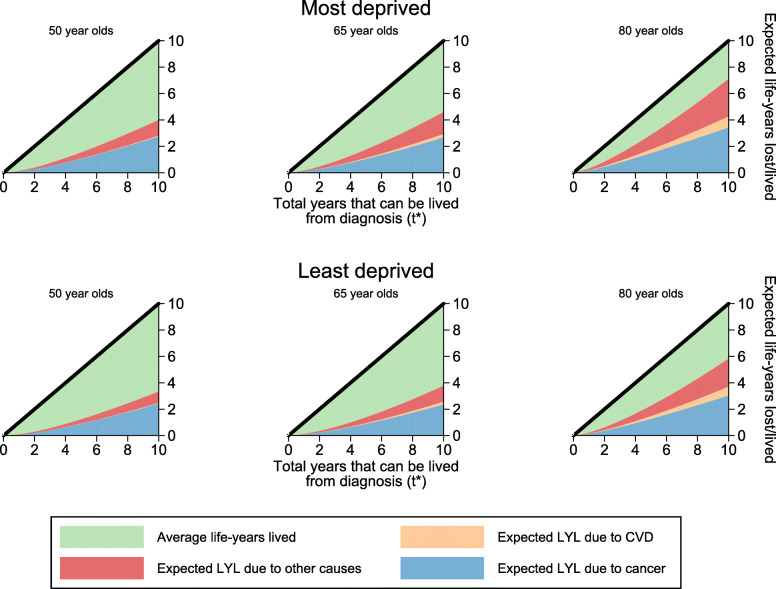
Stacked plots of expected life-years lost partitioned by each cause of death for male patients

As discussed in the Overview of restricted mean survival time for competing risks section, as a useful summary measure, the RMST estimate can be obtained. This is equivalent to the white area of the associated stacked plot in Fig. 1 up to *t*^∗^ for a particular covariate pattern. Conversely, the area of the stacked areas give an estimate of the RMFT. The area of each of the partitioned stacks for each of the respective causes of death yield the expected life years lost due to cancer, CVD and other causes. These are presented for the most and least deprived 50, 65 and 80 year old male patients in Fig. 2. Each of the stacks represent the average life-years lived in total and life-years lost due to a specific cause. The plots here present life-years lost and lived before different points in time up to 10 years from diagnosis. However, particular interest here is in the life-years lived, or lost, *before* 10 years from diagnosis. For example, total average life-years lived before 10 years from diagnosis for the most deprived 50 year old male patients is 3.99 years (95% CI: 3.84 years, 4.14 years). Of the 6.01 years of the total life-years lost, 2.72 years (95% CI: 2.60 years, 2.85 years) are due to cancer, 0.07 years (95% CI: 0.06 years, 0.09 years) are due to CVD and 1.19 (95% CI: 1.11 years, 1.28 years) due to other causes.

Table 1 presents differences in life-years lost due to each cause of death before 10 years from diagnosis between the most and least deprived groups for 50, 65 and 80 year olds, along with their associated 95% CIs. The absolute estimates of expected life-years lost for the most and least deprived patients at the individual ages are also presented. This provides us with an understanding of how many additional life-years most deprived patients are expected to lose due to a specific cause of death in comparison to the least deprived patients. For instance, at 10 years from diagnosis, 50 year old male patients from the most deprived group lose an additional 0.32 (95% CI: 0.28, 0.36) life-years due to cancer, 0.01 (95% CI: 0.01, 0.02) life-years due to CVD and 0.33 (95% CI: 0.30, 0.36) life-years due to CVD compared to the least deprived group. For older male patients aged 80 years old, there is a greater disparity in life-years lost due to CVD (0.16 life-years) and other causes (0.76 life-years) between the most and least deprived.

**Table 1:** Expected LYL for each cause for males aged 50, 65 and 80 years old at diagnosis

	Most Deprived			Least Deprived			Difference		
	LYL	95% LCI	95% UCI	LYL	95% LCI	95% UCI	LYL	95% LCI	95% UCI
50 Yrs Old									
Cancer	2.724	[2.604,	2.848]	2.407	[2.299,	2.519]	0.317	[0.277,	0.357]
CVD	0.069	[0.055,	0.088]	0.056	[0.044,	0.071]	0.014	[0.009,	0.018]
Other causes	1.195	[1.113,	1.282]	0.864	[0.804,	0.929]	0.330	[0.300,	0.361]
65 Yrs Old									
Cancer	2.654	[2.179,	3.232]	2.340	[1.913,	2.864]	0.313	[0.250,	0.377]
CVD	0.271	[0.149,	0.495]	0.219	[0.120,	0.400]	0.052	[0.019,	0.085]
Other causes	1.662	[1.285,	2.149]	1.212	[0.930,	1.580]	0.449	[0.339,	0.559]
80 Yrs Old									
Cancer	3.415	[3.055,	3.818]	3.018	[2.690,	3.386]	0.397	[0.340,	0.454]
CVD	0.840	[0.468,	1.508]	0.681	[0.378,	1.228]	0.159	[0.063,	0.255]
Other causes	2.845	[2.426,	3.337]	2.120	[1.792,	2.508]	0.725	[0.618,	0.833]

### Analysis of data with marginal estimates

When interest is in the covariate effects of particular groups, for example, between deprivation groups, it is useful to obtain standardised estimates as described in the Standardisation for marginal differences section. By marginalising over the same covariate distribution, fairer comparisons can be made between particular covariate groups of interest. In this example, we standardise by age and sex in order to summarise the differences in survival between patients from the most and least deprived groups.

#### Cause-specific probability of death for the Most deprived compared to the least deprived

**Fig. 3:**
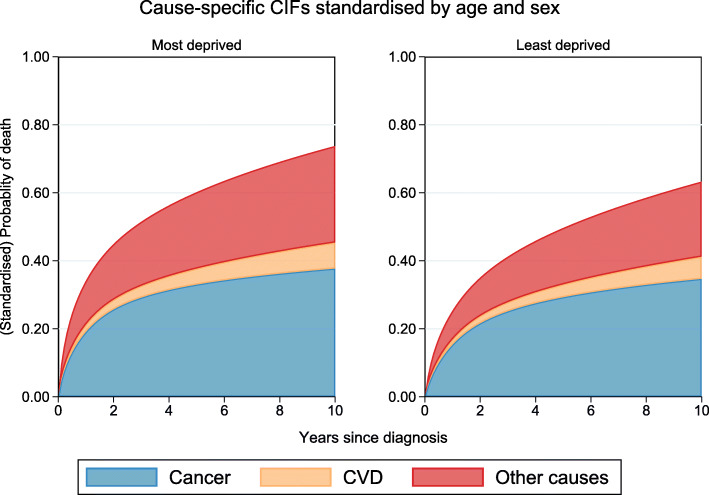
Estimated cause-specific CIFs standardised by age and sex for each deprivation group

**Fig. 4:**
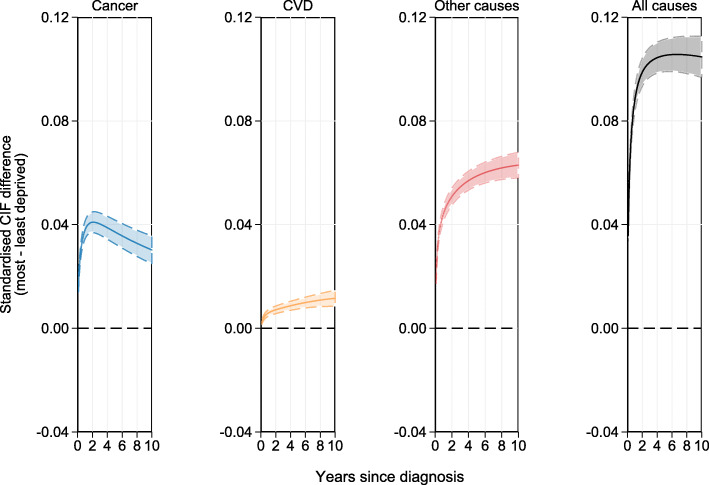
Estimated CIF differences for each cause of death standardised by age and sex with 95% CIs

Figure 3 illustrates standardised CIFs stacked for each cause of death and Fig. 4 presents absolute risk differences for each cause between the least and most deprived patients. As illustrated in Fig. 3, patients from the most deprived group have a higher probability of dying from any cause (73.8%) compared to those from the least deprived group (63.3%). However, when partitioned into the different causes of death, the difference in total mortality between the most and least deprived groups is mostly due to other causes and CVD as indicated by the area proportions. The cause-specific marginal risk difference between the most and least deprived are presented in Fig. 4 along with their respective 95% CIs. As can be seen here, the largest difference in risk is due to other causes and the largest difference in risk between the least and most deprived groups is due to other causes at 10 years from diagnosis (6.3%; 95% CI: 5.8%, 6.9%). Generally, the disparity in the probability of dying from other causes or CVD between the most and least deprived patients continues to increase over follow-up time. However, the cancer-specific risk difference between the most and least deprived increases only for the first 2 years. After this point, the disparity in the probability of dying due to cancer between the most and least deprived begins to decrease.

#### Expected number of life-years lost for the Most deprived compared to the least deprived

**Fig. 5:**
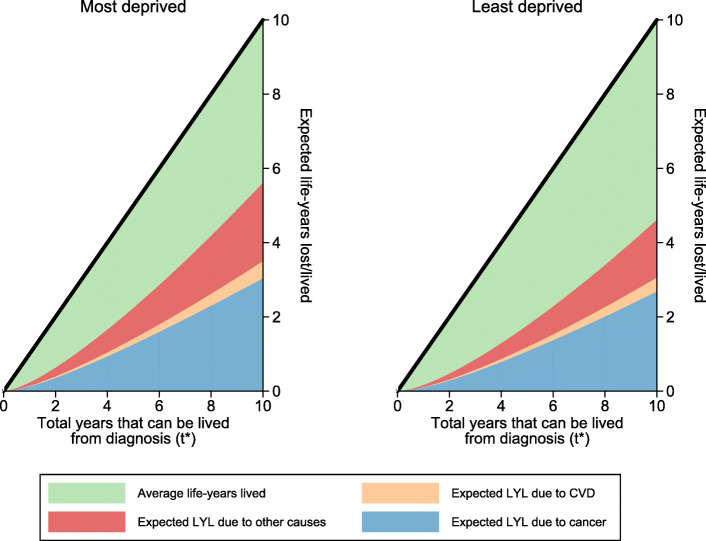
Stacked plots of expected life-years lost for each cause of death standardised by age and sex

In Fig. 2, the expected life-years lost and total average life-years lived were presented for each cause of death before various time-points, *t*^∗^. By obtaining marginal estimates through standardisation over age and sex, we can focus on specific comparisons between the least and most deprived patients. The marginal expected life-years lived for each cause of death and total average life-years lived before each time, *t*^∗^, are similarly illustrated in Fig. 5. If *t*^∗^=10, then we have that the total average life-years lived before 10 years from diagnosis for the most deprived patients is 4.39 (95% CI: 3.78, 5.00). Of the 5.61 total expected life-years lost, 3.03 (95% CI: 2.66, 3.46) years are lost due to cancer, 0.46 (95% CI: 0.27, 0.81) years due to CVD and 2.11 (95% CI: 1.76, 2.53) years due to other causes. By obtaining marginal estimates of expected life-years lost, we are able to directly compare both deprivation groups and determine the additional life-years lost for patients that are the most deprived standardised by age and sex. Thus, where *t*^∗^=10, we have that the additional life-years lost due to cancer, CVD and other causes before 10 years from diagnosis for the most deprived patients is 0.31 (95% CI: 0.25, 0.37), 0.05 (95% CI: 0.02, 0.08) and 0.44 (95% CI: 0.33, 0.54) life-years respectively.

## Discussion

This paper presents novel estimation of RMLT and expected life-years lost from within the flexible parametric survival modelling framework in the presence of competing risks. This can be done either on the CSHs or cumulative incidence scale and allows easy incorporation of time-dependent effects to relax the proportionality assumption. These also offer additional advantages over the more popular Cox PH and Fine and Gray models [5, 7]. In particular, we illustrate how one can easily obtain comparative predictions based on the expected number of life-years lost due to a specific cause of death in addition to other useful estimands, such as absolute differences in the cumulative incidence functions. A common approach for obtaining marginal estimates uses inverse probability weighted estimating equations. However, different estimators need to be calculated subject to whether it is of interest to obtain marginal or non-marginal/conditional estimates [48, 49]. On the other hand, marginal estimates using standardisation are easily obtained in addition to conditional estimates within the FPM approach from a single model. FPMs in both a standard survival analysis and for competing risks data offer numerous advantages in prediction, specifically, through its estimation of the baseline hazard function using RCS and easy inclusion of time-dependent effects. In spite of this, it is also important to consider limitations that are often highlighted. One such limitation is the problem of choosing the appropriate number of knots for the underlying baseline hazard function using RCS, and for when including time-dependent effects when relaxing the proportional hazards assumption. However, a number of extensive simulation studies have been carried out evaluating how many knots are required in order to accurately capture (both simple and complex i.e. time-dependent) shapes of the baseline hazard function. For instance, Bower et. al. [37] and Syriopoulou et. al. [50] both conclude predictions are not sensitive to the choice in the number of knots, provided that a sufficient number of degrees of freedom are used. In other words, too few degrees of freedom may be too simple to accurately capture the effect, and too many will lead to over-fitting. As a guideline, 5 degrees of freedom to capture baseline effects and 3 degrees of freedom for any time-dependent effects are suggested as a starting point. However, it is further suggested that for each individual study, sensitivity analyses are carried out in order to assess model fit and robustness to the choice in degrees of freedom [37, 50]. Syriopoulou et. al [50] also reach similar conclusions with extension to marginal model-based estimates when obtaining predictions using standardisation. Alternatively, a penalised approach for choosing the appropriate number of degrees of freedom for RCS can be used [51]. The interpretation of the RMLT measure also has some notable limitations. Although communication in terms of changes in life-years lost to clinicians and patients rather than probabilities is attractive, applying an upper bound, *t*^∗^, to the time interval may add some difficulty in understanding of the measure. This is because, RMLT for an arbitrary choice of *t*^∗^ can only be used to estimate the average risk within a restricted time period for a group of patients. Furthermore, it should be highlighted that the expected life-years lost makes comparison with an immortal cohort where patients are alive for the whole interval from 0 to time *t*^∗^. A similar “unrestricted” measure that do not compare to an immortal cohort can be estimated within the relative survival framework based on extrapolation of the excess hazard rate. This is usually referred to as the number of life years lost, or the loss in expectation of life and is calculated based on a comparison of the life-expectancy of cancer patients to a comparable population group who are assumed to be cancer-free [52–54]. However, this relies on the assumption that this extrapolation is appropriate which is not made for the RMLT estimate. In addition to the above, due to the dependence of the interpretation of RMST on follow-up time, comparison between different studies, for example, between countries, becomes difficult. It has also been further shown that the difference in RMST between two covariate groups depends on the outcome rates within each group. Therefore, it is recommended that differences in RMST, RMFT and expected number of life-years lost, are reported alongside their respective survival, or cumulative incidence functions, in order to allow comparability and to obtain the entire picture of the impact of different groups on outcome [24]. This further points to additional advantages of estimation of RMFT within the flexible parametric modelling framework, as these additional measures are easily obtained from the same model.

## Conclusions

The RMLT measure is presented as a useful summary measure with an attractive interpretation which can aid in the analysis of competing risks data. As discussed by others, it is also useful to present estimated cause-specific CIFs alongside CSHs [6, 34]. We propose FPMs as the chosen estimator as it allows easy estimation of various estimands from a single model providing both conditional and marginal estimates. Note that, although not discussed here, if appropriate confounders are adjusted for, one can also infer causal effects between two groups using standardisation. However, one must also consider the additional complexities and issues in interpretation with the inclusion of time-dependent risk-groups [44]. Furthermore, the RMLT measure can be easily extended for obtaining conditional estimates, for example, the average life-years lived before *t*^∗^ years given survival to time *t*_0_ from diagnosis. Example Stata code for the model and prediction of measures provided in this paper is outlined in Appendix: Stata code for obtaining predictions.

## Appendix A: Restricted cubic spline variables

Given a vector of *M* knots, **m** and a vector of *M*−1 parameters, *γ*, with *M*−1 degrees of freedom (df), the restricted cubic spline function, *s*(ln(*t*);*γ*,**m**), is defined as,

31 $$ s(\ln(t); \pmb{\gamma}, \mathbf{m}) = \gamma_{0} + \gamma_{1}z_{1} + \cdots + \gamma_{(M - 1)}z_{(M - 1)}  $$

Where *z*_1_,⋯,*z*_(*M*−1)_ are the basis functions of the restricted cubic splines and are defined as,

32 $$ z_{1} = \ln(t)  $$

$$\begin{array}{*{20}l} {}z_{j} &= (\ln(t) - m_{j})_{+}^{3} - \phi_{j}(\ln(t) - m_{1})_{+}^{3}\\ &- (1 - \phi_{j})(\ln(t) - m_{M})_{+}^{3}, & j &\,=\, 2, \cdots, M - 1 \end{array} $$

where,

33 $$ \phi_{j} = \frac{m_{M} - m_{j}}{m_{M} - m_{1}}  $$

and

34 $$ (u)_{+} = \begin{cases} u,& \text{if}\ u > 0\\ 0, & \text{otherwise} \end{cases}  $$

Usually, *M* knots are placed at equally spaced centiles of the distribution of the uncensored log-survival times including two boundary knots at the 0^*th*^ and 100^*th*^ centiles.

## Appendix B: Gaussian quadrature

With the general Gaussian quadrature rule, the integral of any polynomial function, *g*(*u*), over the interval [−1,1] can be evaluated. This performs best for integrals that can be approximated by a polynomial function of degree 2*m*−1, where *m* is a pre-determined number of points, otherwise known as nodes, or abscissae. Hence, this integral can be evaluated for,

35 $$ \int_{-1}^{1} g(u) \text{d}u = \int_{-1}^{1} W(u)g(u) \text{d}u  $$

where, *W*(*u*), is a known weighting function. Here, the integral, e.g. the cause-specific cumulative incidence function, is calculated using Gauss-Legendre quadrature, with *W*(*u*)=1. With this, based on a set of pre-defined number of nodes, $u^{\prime }_{i}$, and associated Lagrange polynomials of degree *m*,*P*_*m*_(*u*), weights, $w^{\prime }_{i}$, for *i*=1,…,*m*, are obtained such that,

36 $$ w^{'}_{i} = \frac{2}{(1-u^{'2}_{i}) \left(P'_{m}(u'_{i})\right)^{2}}  $$

and are provided by Abramowitz and Stegun [55]. Therefore, Eq. 35 is approximated by,

37 $$ \int_{-1}^{1} g(u) \text{d}u \approx \sum_{i = 1}^{m} w'_{i} g(u'_{i})  $$

However, for survival data, functions are evaluated over an interval [0,*t*]. Therefore, to apply the Gaussian quadrature rule in Eq. 35, integrals over the interval [0,*t*] must be changed to an interval over [−1,1] such that,

38 $$ \int_{0}^{t} g(u) \text{d}u = \frac{t - 0}{2} \int_{-1}^{1} g\left(\frac{t - 0}{2}u + \frac{t + 0}{2}\right) \text{d}u  $$

Therefore, a function evaluated at *t*_1_,…,*t* different time-points over an interval [0,*t*] is approximated by applying Gaussian quadrature rules with *W*(*u*)=1 such that,

39 $$ \int_{0}^{t} g(u) \text{d}u \approx \frac{t - 0}{2} \sum_{i=1}^{m} w'_{i} g\left(\frac{t - 0}{2}u'_{i} + \frac{t + 0}{2}\right)  $$

## Appendix C: Additional summary statistics

Table 2 provide summary statistics on the distribution of key covariates of interest for inclusion in analysis i.e. sex, deprivation group (least/most deprived) and age, by cause of death, and in total.

**Table 2:** Distribution of data on key covariates included in the analysis for n = 159,022 patients

	Females, n(%)	Least deprived, n(%)	Age, mean(sd)
Cancer	21 137 (43.27)	25 084 (51.35)	72.25 (10.57)
CVD	3 158 (39.54)	3 853 (48.24)	76.78 (7.96)
Other Causes	13 716 (42.71)	14 955 (46.57)	74.04 (9.64)
All Causes	38 011 (42.74)	43 892 (49.35)	73.30 (10.13)
Alive/Censored within 10 yrs	30 663 (43.76)	43 079 (61.47)	68.05 (9.97)
Total	68 974 (43.19)	86 971 (54.59)	70.99 (10.39)

**Fig. 6:**
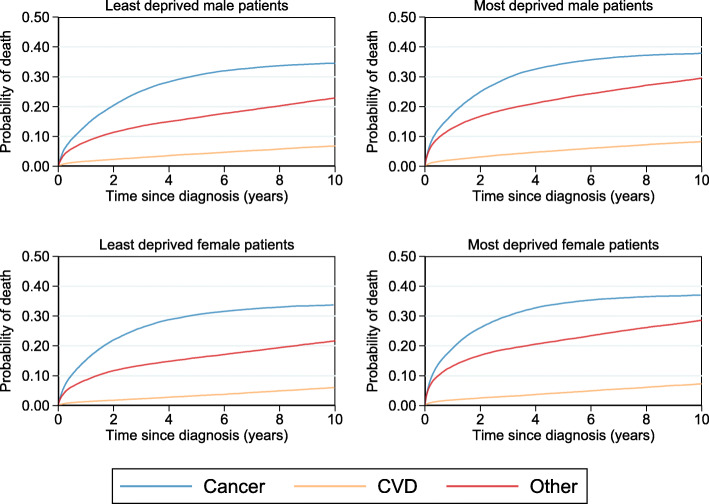
Cause-specific cumulative incidence functions (CIFs) Aalen-Johansen estimates for each cause of death

Figure 6 represents the cause-specific cumulative incidence functions estimates obtained by the non-parametric Aalen-Johansen estimator. This summarises the probability of dying from each cause of death by sex and deprivation groups.

**Fig. 7:**
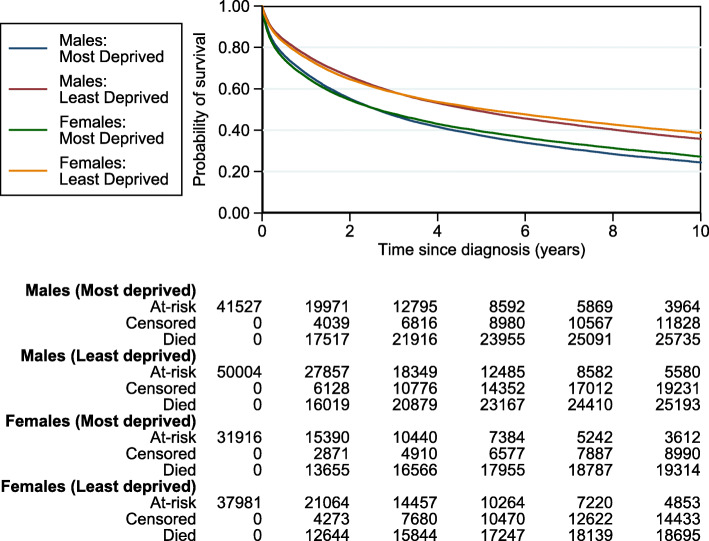
All-cause Kaplan-Meier survival probabilities by sex and deprivation groups

Figure 7 illustrates the all-cause survival probabilities obtained by the non-parametric Kaplan-Meier estimator. This summarises the all-cause probability of survival by sex and deprivation groups.

### Stata code for obtaining predictions

This appendix outlines Stata code used to obtain predictions presented in the paper. Some user-defined Stata commands are required which can be installed from the Boston College Statistical Software Components (SSC) archive by calling,


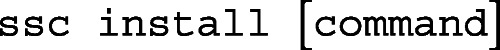


The following must be installed before running the code:

- stpm2: To fit the flexible parametric models described in Flexible parametric survival models section.
- rcsgen: To generate the restricted cubic spline functions.
- stcrprep: To restructure data and calculate time-dependent censoring weights in order to fit models on the subdistribution hazards scale using standard Stata commands.

To obtain marginal (and non-marginal) estimates using standardisation, the standsurv command must be installed. This will be released on SSC soon, however, in the meantime, it can be installed by running,


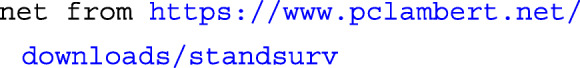


#### Preparing the data for analysis

To prepare the data for a survival analysis in Stata, we must first run the stset command. We identify the variable that records survival time (in days), exit2, the indicator variable for cause of death, cod, where death from cancer = 1, CVD = 2 and other causes = 3 and finally the variable for date of diagnosis, dx. The scale option is used to transform the survival time into years from days and we use the exit option to restrict follow-up time to 10 years from diagnosis and censor those still alive at 2014. In order to ensure that the death indicator, _d, generated after stset matches the death indicator for cause of death, we create a new cause of death indicator, cod2, so that those who die either after 10 years from diagnosis or 2014 are administratively censored. Finally, to generate restricted cubic spline variables for the non-linear effect of age centred at 45 years old at diagnosis, we use rcsgen. For 3 degrees of freedom, 3 new age spline variables are created, rcsage1 − rcsage3, and we store knot positions and matrix for orthogonalization which are required for post-estimation predictions at specific ages.


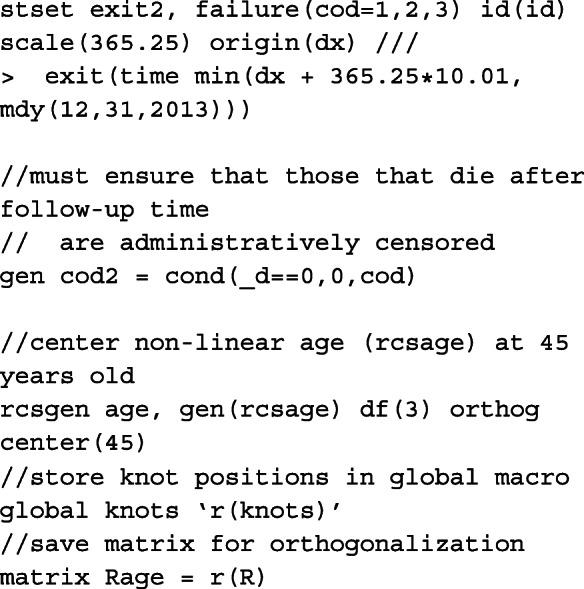


To restructure the data and calculate the time-dependent censoring weights so that we may fit a model on the subdistribution hazards scale, we use stcrprep[56]. Here, we specify wtstpm2 to estimate the censoring distribution using a Royston-Parmar flexible parametric model with covariates included in the censcov option. The data is restructured based on the variable failcode, which splits the data according to the cause of interest. This is used to fit identify for which cause the model is to be fitted for. For clarity, we create dummy variables for each of the causes of death from failcode and generate _cancer, _cvd and _other. Another indicator variable, event, is also created to identify at which split time interval, or row, death (from any cause) is observed for that patient. To incorporate the calculated weights from stcrprep, we must stset the data again with tstart and tstop. These are also provided by stcrprep and give the times at which an individual starts and stops being at risk.


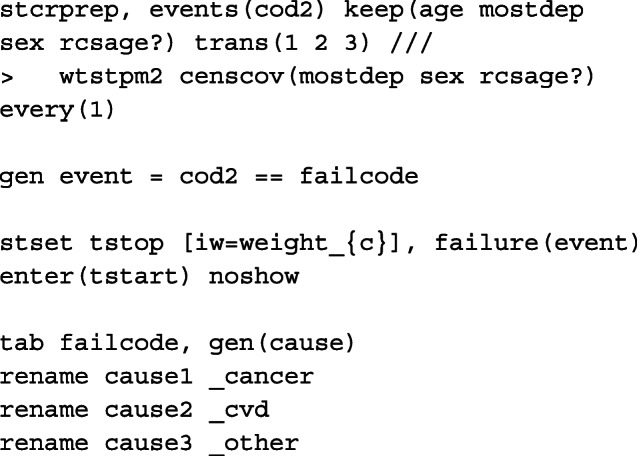


#### Model

The model described in Flexible parametric survival models section can be fitted in two ways after preparing the data. We can either fit separate models for each of the causes of death, or fit a single model to cancer, CVD and other causes simultaneously. Here, we demonstrate for the latter to make illustration of the code for obtaining predictions post-estimation easier. However, in order to fit the equivalent single model with coefficients comparable to the models fitted individually to each of the causes of death, the knot locations on the cause-specific survival time distributions must be stored. These are stored in global macros for each of the causes of death.


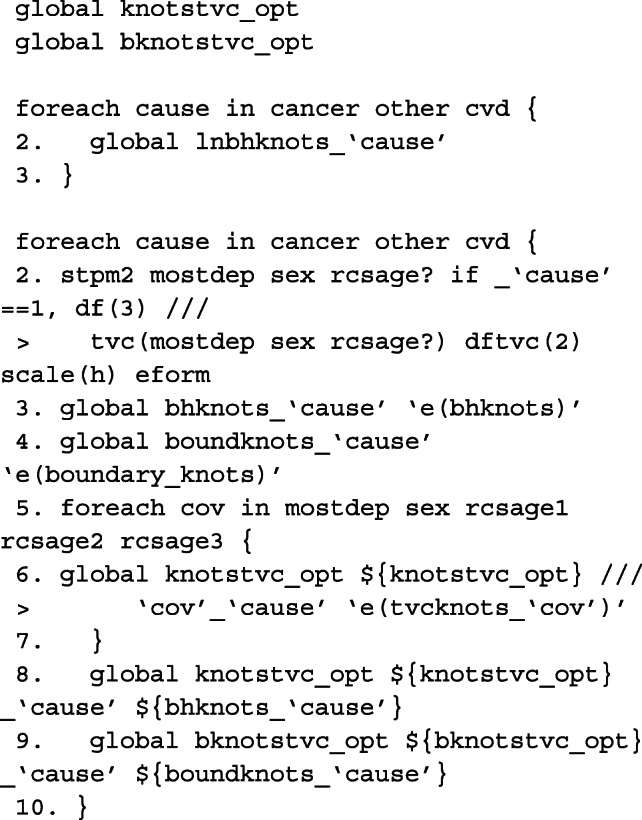


Here we define a global macro of the list of covariates to be included in the single model. As the data is stacked, interactions need to be created between the covariates and the indicator variable for each cause of death. See Lunn and McNeil[57] for further details. The baseline coefficient, i.e. the constant in the cause-specific model, is calculated in _cancer, _cvd and _other. We therefore fit a model for each of the causes of death simultaneously without a constant using nocons and the baseline splines using rcsbaseoff. Instead, the baseline splines are specified as time-dependent splines for the coefficient that corresponds to the constant in its respective model for that particular cause of death. These were stored in the global macro bknotstvc_opt. Since knots are specified according to the time scale, rather than the log-time scale, the knscale(time) option is used.


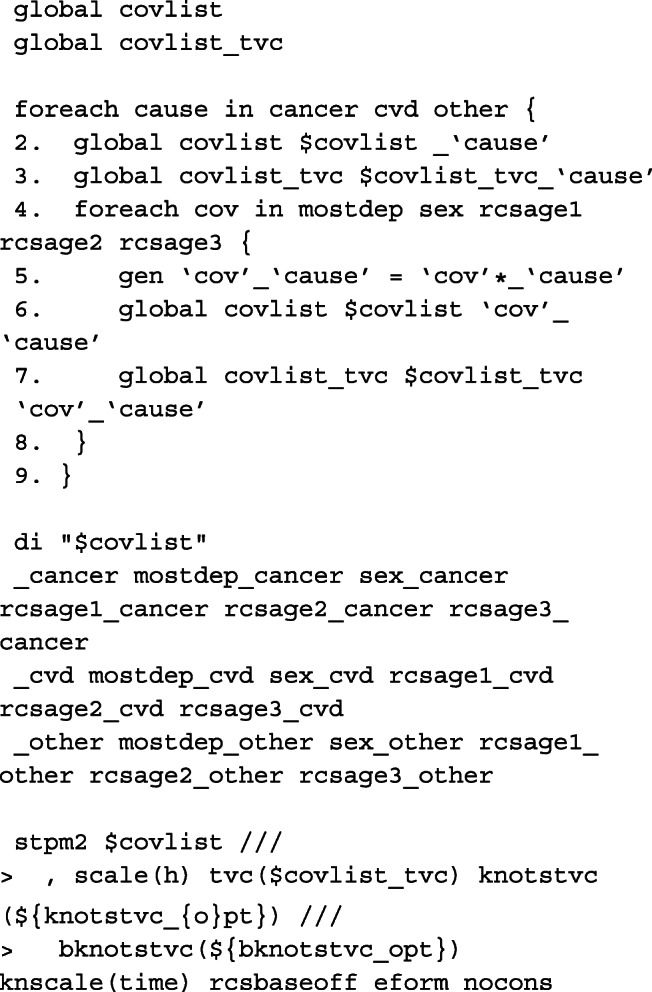


#### Predictions

Although standsurv was written for obtaining marginalised predictions, it can also be used to obtain non-marginalised estimates. This is done by simply specifying the entire covariate pattern so that the predictions are not averaged over any covariate distribution. To obtain predictions at a specific age, we need to calculate the spline variables at that particular age centred at 45 years old with the same knot locations and projection matrix as before. The spline variables are stored in the local macros c1, c2 and c3. An example is given below when the cause of interest is cancer and we want to make comparisons between the most and least deprived male patients aged either 50, 65, or 80 years old at diagnosis.


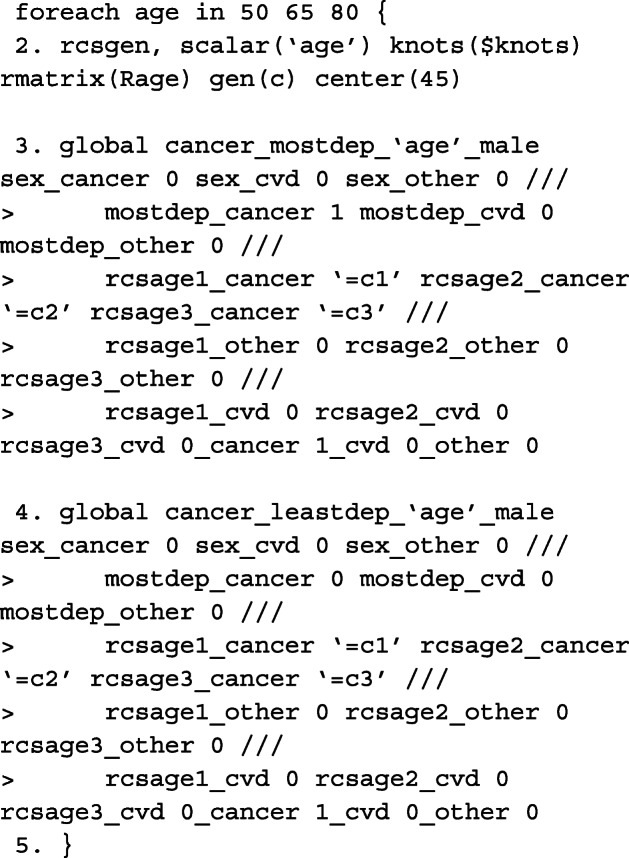


As we do not average over each observation, we must tell standsurv to only take the first observation in the stacked data to calculate non-marginalised predictions. This is done using if _n == 1. The failure option is used to obtain the cumulative incidence functions that is specified in each at option. To calculate the difference between at1 and at2, we use contrast(difference).


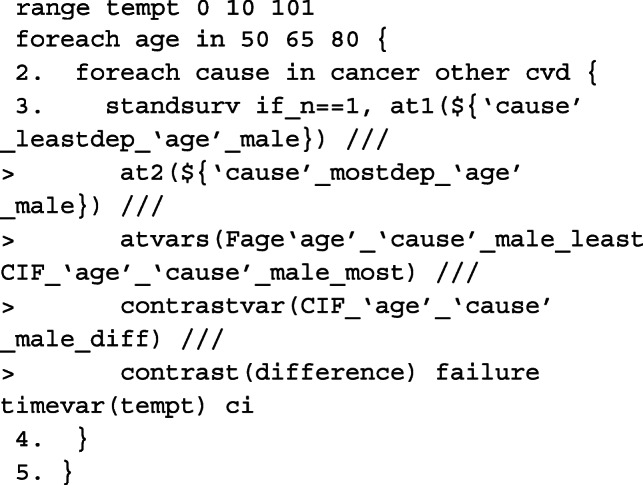


Since we are making predictions at particular covariate patterns for each of the causes separately, specifying rmft gives us estimates of the expected life-years lost due to a particular cause of death. To calculate RMLT, we need to take the sum of all of the at options, where the expected life-years lost due to cancer, CVD and other causes is specified in each. We do this by creating our own contrast in a user-defined mata function which can be called in the option userfunction. An example of this is also given below.


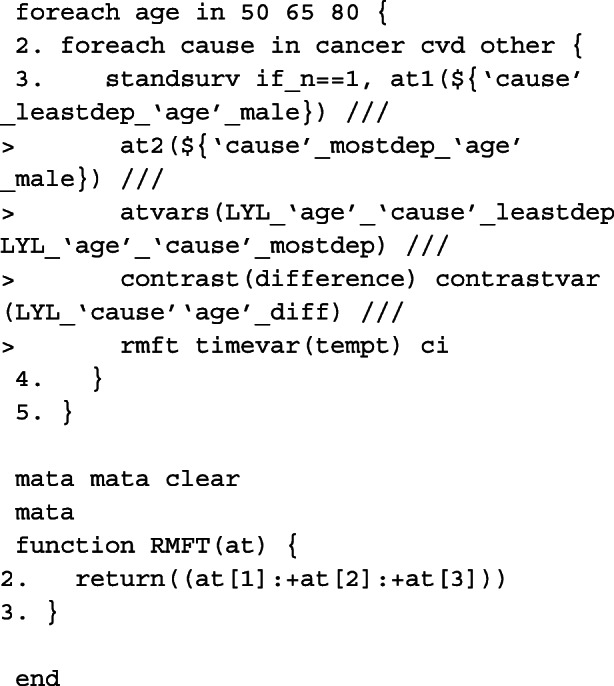


In order to obtain marginalised estimates, in each at option, only the covariate pattern for the group of interest need to be given. For the covariate distribution that we want to average over, as we have created interactions between the covariates and the causes of death, these must be mapped to each covariate e.g. sex_cancer = sex. The others are excluded from the at option for the other causes of death. In this case, because we want to average over covariates that we wish to standardise by, we need to identify the row for each patient in the stacked data that corresponds to the failure time of that individual. This is done by creating the indicator variable first and using it as an if condition in standsurv. As before, we give an example for specifying macros for use in the at options for deaths due to cancer.


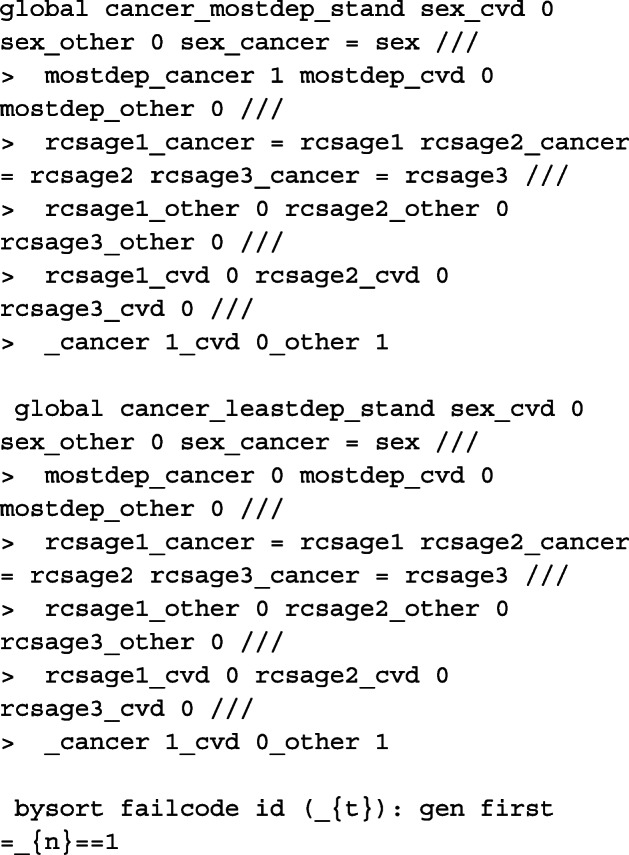


The cause-specific CIF differences are thus calculated as follows,


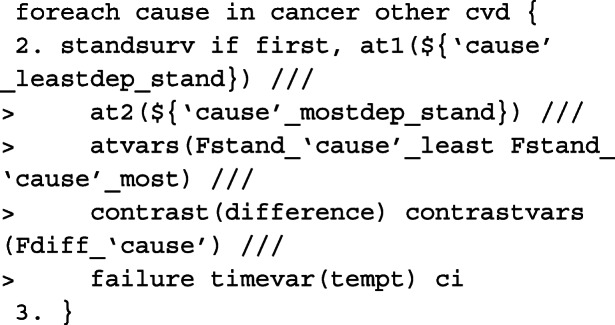


As highlighted above, we can write user-functions to define our own contrasts. Below is an example for when interest is in calculating the difference in RMLT between the most and least deprived patients.


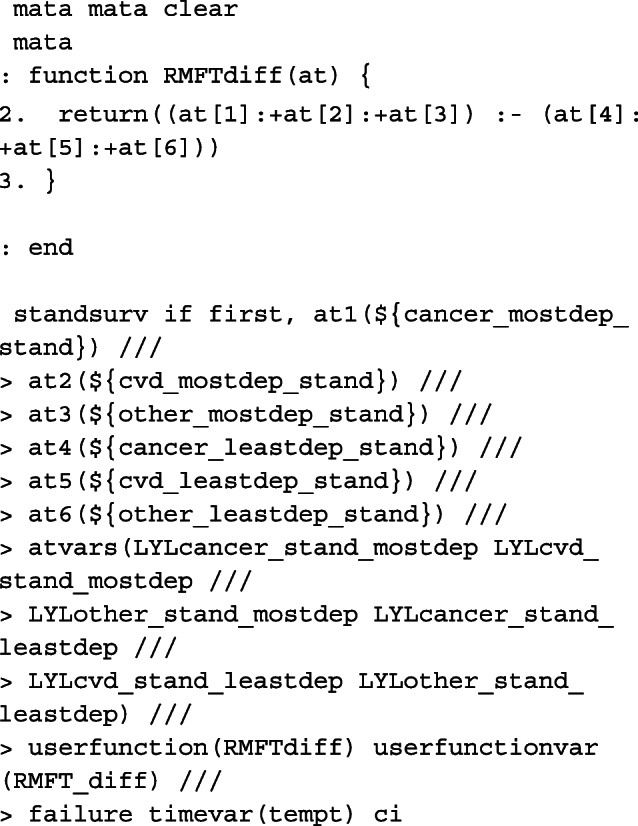


## Data Availability

The data that support the findings of this study are available from Public Health England (https://www.gov.uk/government/publications/accessing-public-health-england-data/about-the-phe-odr-and-accessing-data), but restrictions apply to the availability of these data, which were used under license for the current study, and so are not publicly available.

## Abbreviations

CSH: Cause-specific hazards
CIF: Cumulative incidence function
CI: Confidence interval
CVD: Cardiovascular disease
DF: Degrees of freedom
FPM: Flexible parametric model
FPCIM: Flexible parametric cumulative incidence model
HR: Hazard ratio
LYL: Life-years lost
RCS: Restricted cubic splines
RMFT: Restricted mean failure time
RMLT: Restricted mean lifetime
RMST: Restricted mean survival time
SDH: Subdistribution hazards
SHR: Subdistribution hazard ratio
